# Tailoring the surface pore morphology of bioceramic scaffolds through colloidal processing for bone tissue engineering

**DOI:** 10.1371/journal.pone.0318100

**Published:** 2025-02-27

**Authors:** Shareen S. L. Chan, Daniel E. Heath, George V. Franks

**Affiliations:** 1 Chemical Engineering, Melbourne School of Engineering, University of Melbourne, VIC, Australia; 2 Biomedical Engineering, Melbourne School of Engineering, University of Melbourne, VIC, Australia; Universidade de Trás-os-Montes e Alto Douro: Universidade de Tras-os-Montes e Alto Douro, PORTUGAL

## Abstract

In this study, porous bioceramic scaffolds are developed with two materials, β-tricalcium phosphate (β-TCP) and hydroxyapatite (HA), with order of 10 micron-scale surface pores and approximately 40–60% volume porosity fabricated by soft templating of oil. Suitable oil and surfactant concentrations are determined for the creation of particle-stabilized emulsions with nearly spherical pores, as well as the capillary suspensions with elongated pores. The bioceramic scaffolds surfaces are then assessed for their ability to support osteoblast adhesion and growth, for applications as scaffolds for bone regeneration. The porous scaffolds’ surfaces are compared to denser surfaces of the same material, where only submicron porosity arise from partial sintering, to interrogate the impact of surface topography on cell behavior. On the denser surfaces where no large pores are templated, β-TCP supports a larger number of osteoblasts compared to HA. Templated surface porosity significantly impacts the morphology and growth of the osteoblasts. Amongst the pore morphologies, the capillary suspension demonstrates enhanced biological function, whereas the emulsion performs the poorest. The β-TCP capillary suspension scaffold surface appears to provide the most favorable conditions for the osteoblasts.

## 1. Introduction

Global deaths and disability-adjusted life-years (DALYs) attributed to fractures from bone diseases (such as osteoporosis and low bone mass) have more than doubled in the last thirty years [1], exerting serious economic and social burdens on populations worldwide [2]. In orthopaedic repair and replacement, allografts and autografts currently constitute the “gold standard”. However, they are associated with shortcomings such as immunogenic rejections and limited availability [3]. With an improving life expectancy translating to an expected increase in musculoskeletal diseases and injuries worldwide, it is imperative to develop high-performing synthetic bone substitutes to meet the demand [4].

The role of synthetic bone substitutes has fundamentally shifted from tissue replacement to tissue regeneration, tapping into our ability to self-heal with a 3D scaffold that provides temporary mechanical support for physiological loads, as well as stimulates cell growth [5]. Currently, metals are used for repairing load-bearing bone defects such as in bone fixation plates and screws [6, 7], while polymers (including hydrogels) are the most commonly 3D-printed material (86%) for medical applications [8]. Although they are biocompatible and mechanically stable, they are bioinert and either have limited bioresorptive capabilities [9] or produce acidic degradation products which increase the risk of inflammation [10–12]. Ceramics such as hydroxyapatite (HA) and beta-tricalcium phosphate (β-TCP) closely resemble the mineral component of natural bone and provide excellent biocompatibility as well as better bioactivity. These calcium phosphates have demonstrated good bone ingrowth in non-critical-size defects most likely due to their superior bioresorbability [13]. Their ability to dissolve over time, releasing calcium and phosphate ions, is believed to be their source of bioactivity [14]. On the other hand, ceramics are inherently brittle and have limited load-bearing abilities. Dense calcium phosphates have a Young’s modulus of 40–117 GPa [15], which is higher than the reported range of 0.02–5 GPa for trabecular bone [16]. The elastic modulus for medical beta titanium alloys is in the range of 14–85 GPa [17]. It is important to match the elastic modulus of the implant to the surrounding bone otherwise stress shielding may occur, which is a common issue with conventional metallic implants [18, 19]. However, it may be possible to overcome these issues by producing multiscale porous structures that have a lower modulus and enhanced blunting of crack tips when encountering pores [20]. This is the primary reason why dense hydroxyapatite and TCP are not typically used in bone healing and regeneration applications, except when in granular configuration. Instead, porous ceramics with modulus similar to trabecular bone are typically used. This is the approach we apply in the current research.

**Fig 1:**
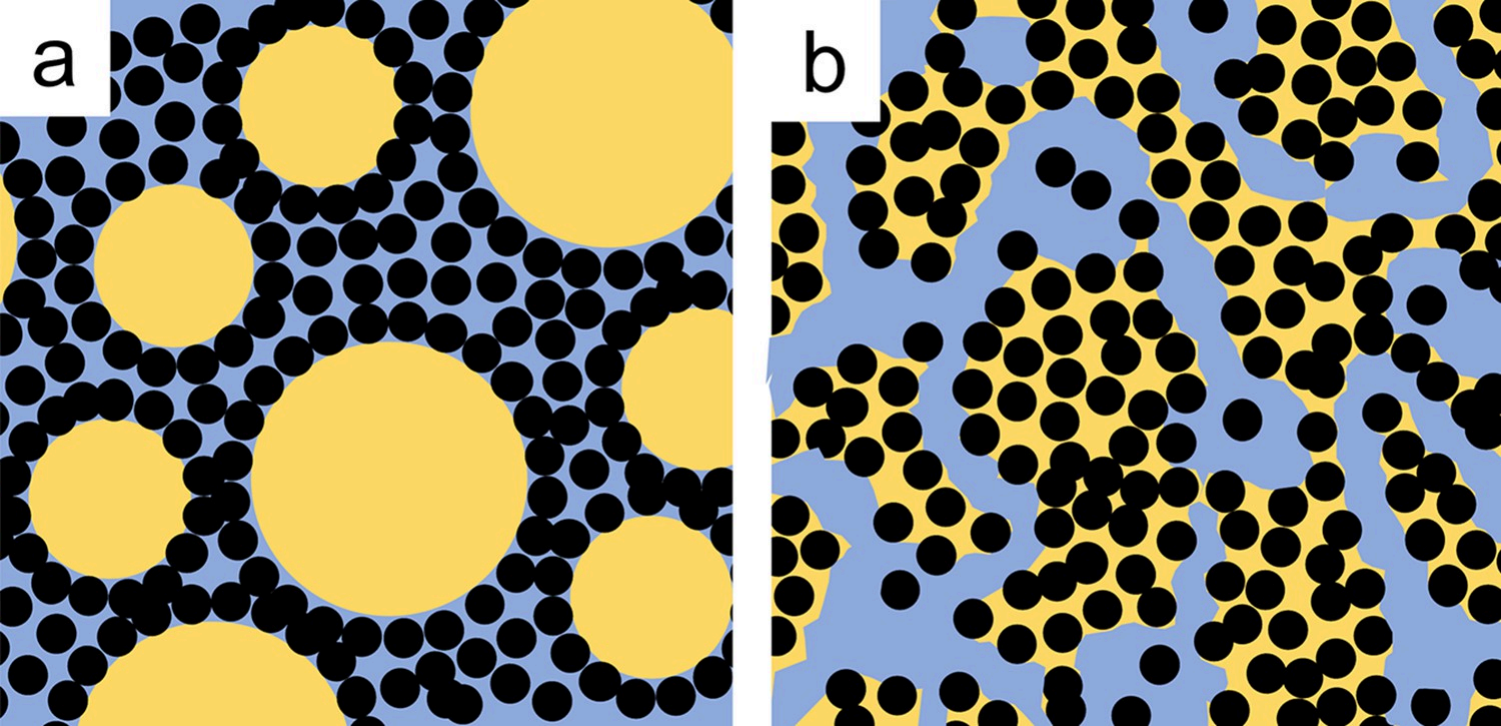
Schematic illustration of (a) particle stabilized emulsions where weakly hydrophobic particles (black) stabilize oil droplets (gold) in an aqueous (blue) suspension of particles and (b) capillary suspensions where strongly hydrophobic particles produce a bicontinuous interconnected two-phase oil-water system, where perhaps the particles partially or completely transfer into the oil phase. (Note this description of the capillary suspensions is slightly different from our previous hypothesis published as Fig 1 in reference [39]).

The ability to use porous ceramic formulations to better match the mechanical properties of native bone tissue is a promising avenue of research. However, cells are highly sensitive to the physical cues in their micro-environment, including surface roughness and topographical features. For instance, cell culture experiments have shown that microscale and nanoscale features, such as surface roughness, grooves, or nanopatterns can enhance cell adhesion, proliferation, and differentiation. These features have been created using techniques such as grinding [21, 22], electron beam irradiation [23], anodization and chemical etching [24], however such techniques are generally limited to accessible surfaces. An interconnected, open porous structure is advantageous for the transport of nutrients and metabolites. In other words, fluid permeability is essential for improved bone ingrowth [25]. There are many approaches to producing internally connected porosity in ceramics [26–28], such as solid porogen leaching [29] and freeze-casting [30, 31]. Porogens are referred to herein as a sacrificial material included within the main structure to form pores after it is removed, for example by pyrolysis or dissolving. Some of their shortcomings respectively include smaller and fewer pores on the surface than in the interior [29], and difficulty achieving homogeneity throughout the scaffold [32]. Other techniques to create open cellular networks include foam impregnation molding, however during calcination of the foam template, cracks often appear [33]. In this work, we apply a soft porogen templating technique with the purpose of fabricating a homogeneous interconnected porous structure. Interconnected open-cell pore structures created by soft porogen templating has also been reported for materials such as alumina [20, 40, 86], hydroxyapatite [81], without issues reported of cracking. The specific goal of this research is to understand the various types of porous ceramic structures that can be generated via oil templating, and to understand the impact of the surfaces of these structures on osteoblast cell behaviour. This work is important because the fundamental interaction of cells with the material must be established so that appropriate formulations can be selected for device fabrication. Oil was used as the pore forming agent instead of air, due to the smaller oil droplet sizes achievable than air bubbles [34]. The oil, binder and surfactants are completely burned out during sintering of the material at 1100°C. Therefore, only the ceramic would be left in the scaffolds, so the scaffolds are expected to be biocompatible. Micropores smaller than 10 μm have been shown to enhance protein adsorption and ion exchange [35]. The authors have previously demonstrated with oil-templated alumina-stabilized emulsions that micropore size and porosity can be controlled by modifying formulation parameters, such as the concentrations of oil and surfactant, particle size and mixing speed [40]. Other researchers have also proven the controllability of the pore formation process in a variety of ceramics by particle stabilization of oil droplets [26, 27, 34, 48]. In this study, we create two types of structures. The first is the particle stabilized emulsion (or Pickering emulsion), and the second is another structure which may be the capillary suspension or bijel structure [36–38], but we will refer to it here as capillary suspensions. These two structures produce two different surface pore morphologies. A schematic illustration of these two microstructures is shown in Fig 1. We then investigate the *in vitro* osteoblast performance on three materials and two surface microstructures.

## 2. Materials and methods

### 2.1 Materials

β-tricalcium phosphate (β-TCP) and Hydroxyapatite (HA) was obtained from Plasma Biotal Limited, UK. Polycaprolactone (PCL) with number-averaged molecular weight of 80,000 was obtained from Sigma Aldrich, as a control. The particle size distribution of the ceramic powders (dispersed into RO water) was measured using a laser diffraction particle size analyzer (Mastersizer 3000, Malvern, UK). A refractive index of 1.63 and an absorption index of 2 were utilized. Before measurement, ultrasound of 20 W was applied to the suspension for 1 min, as well as mechanical stirring at 1000 rpm (Hydro EV, Malvern, UK). Five measurements were taken per replicate, and a total of three replicates were measured. The size statistics of the particles and their distributions are presented in the supplementary materials. The d_50_ of the β-TCP is 1.5 μm and the HA is 3.0 μm.

Polyvinyl alcohol (PVA) was obtained from Sekisui Speciality Chemicals as Selvol205S, for use as a binder. Cetyltrimethylammonium bromide (CTAB) was obtained from Sigma-Aldrich, for use as a surfactant. Sunflower oil (Dhara sunflower oil, Parsram Foods Pty Ltd, Malaysia) was used as the soft porogen.

### 2.2 Preparation of colloidal ceramic suspensions

**Fig 2:**
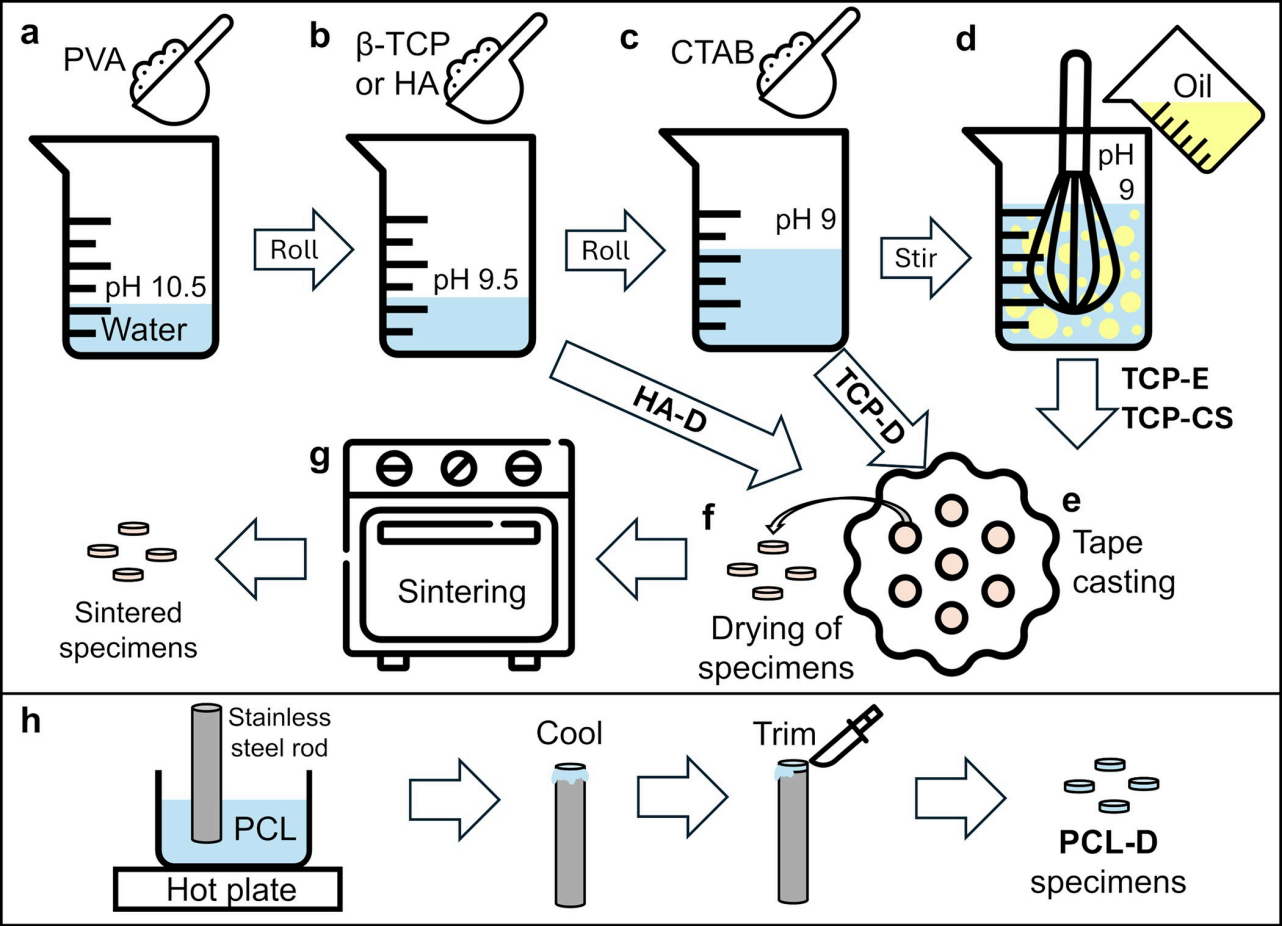
Schematic illustration of processing procedures, presenting (a) addition of polyvinyl alcohol (PVA) to water, (b) addition of ceramic powders to the PVA solution, (c) addition of surfactant cetyltrimethylammonium bromide (CTAB) to TCP suspensions, (d) thin slip casting, (f) drying and (g) lastly sintering of specimens. HA-D undergo steps a, b, e, f and g; TCP-D undergo steps a, b, c, e, f and g; TCP-E and TCP-CS undergo all steps a to g. (h) Procedure for the preparation of PCL specimens by dip-casting, showing the dipping of a stainless steel rod into a PCL melt pool, cooling and trimming of specimens to size. Colours and dimensions used in this schematic are not representative and are only for illustrative purposes.

Please refer to Fig 2A–2A for a schematic of this procedure. Polyvinyl alcohol (PVA) (4 wt.% by solids weight), used as a binder for improved green strength, was dissolved in MilliQ water (Fig 2A). The pH was adjusted to 10.5 with sodium hydroxide, then rolled overnight. An aqueous suspension was made by stirring in 35 vol.% β-TCP powder into the alkaline PVA solution (Fig 2B). The ceramic suspension was adjusted to approximately pH 9.5 with sodium hydroxide. To achieve pH equilibrium and a homogeneous suspension, it was rolled overnight. A final adjustment to pH 9 was conducted before the addition of surfactant. Surfactant, as well as sunflower oil, were added to these suspensions to produce either particle-stabilized emulsions or capillary suspensions similar to those previously reported [39, 40] and described in Fig 1. Cetyltrimethylammonium bromide (CTAB) was obtained from Sigma-Aldrich and added as a weight percentage of total solids (Fig 2C). As shown below, the 0.04 wt.% produced particle stabilized emulsions referred to as TCP-E, while the 0.06 wt.% produced capillary suspensions referred to as TCP-CS. For the denser β-TCP sample where only submicron porosity resulted from partial sintering (henceforth denoted as TCP-D), 0.06 wt.% CTAB was added to increase its viscosity. For the dense hydroxyapatite sample (henceforth referred to as HA-D), the above protocol applies except that a lower solids fraction of 20 vol.% was used and no surfactant was added.

### 2.3 Preparation of particle-stabilized emulsions and capillary suspensions

Oil templating was used to increase the amount of porosity in the ceramic materials. The ceramic suspension was stirred with a whisk coupled to an overhead mixer (RZR 2020, Heidolph, Germany) at a speed of 400 rpm (Fig 2D). Sunflower oil (Dhara sunflower oil, Parsram Foods Pty Ltd, Malaysia; density = 0.90 g/cm^3^) was slowly added into the suspension, over a period of 5 min. The amount of oil, as a volume percentage of total emulsion or capillary suspension, was 40 vol.% for the emulsion and 60 vol.% for the capillary suspension.

### 2.4 Thin slip casting

The ceramic pastes were spread out on a slab of plaster of Paris and the top surface flattened out with a blade to a height of about 5 mm. Molds of 12 mm diameter were used to cut out cylindrical samples (Fig 2E).

### 2.5 Sintering

Samples were dried in air for more than 24 h under ambient conditions (Fig 2F). To avoid warpage of the samples, no additional steps were undertaken to remove the oil phase before sintering. Pressureless sintering in air was conducted in an electrical furnace (Model 1700, Tetlow Kilns & Furnaces Pty Ltd, Australia). The temperature increase was set at 5°C/min and the ramp down at 10°C/min. Additives were pyrolyzed at 350°C for 1h, followed by sintering at 1100°C for 1h (Fig 2G).

### 2.6 Preparation of PCL samples

Please refer to Fig 2H for a schematic of the preparation procedure. Polycaprolactone (PCL, Sigma Aldrich) with number-averaged molecular weight of 80,000 was used as a control for comparison with the ceramic samples. The PCL pellets were melted, then a stainless-steel rod with a flat end of 12.5 mm diameter was dipped in. The dip-cast PCL samples cooled and solidified in air at room temperature, before being detached and having any edges removed.

### 2.7 Density measurements

All measurements were conducted on sintered cylindrical samples. Open porosity refers to pores which are accessible by water from the exterior of the sample, whereas closed pores are completely surrounded by solid without necks to the exterior. “Dry weight” refers to the mass of the sample in air. The samples are then boiled in water for about 30 minutes to allow water to penetrate any open pores. After boiling and cooling down, the sample is weighed while submerged in water, which is the “submerged weight”. After removing the sample from the water and gently blotting off excess surface water, the sample is weighed again in air, which is the “saturated weight”. The Archimedes’ apparent density (*ρ*_*A*_) was measured by the Archimedes’ principle from the dry and submerged weights (see Eq 1), and considers only the volume of the solid and closed pores. The Archimedes’ bulk density (*ρ*_*B*_) was calculated from the dry, submerged and saturated weights (see Eq 2), therefore it also includes the volume of open pores. Total porosity is determined from the Archimedes’ bulk density (*ρ*_*B*_) and the theoretical density (*ρ*_*TD*_) of β-TCP at 3070 kg/m^3^ and hydroxyapatite at 3160 kg/m^3^ (see Eq 3). Open porosity is calculated from the dry, submerged and saturated weights as per Eq 4. Closed porosity is the total porosity less the open porosity (see Eq 5). 5 replicates were measured for the Archimedes method.


$$\rho_{A}=\frac{Dry\;weight}{Dry\;weight-Submerged\;weight}$$ 1

$$\rho_{B}=\frac{Dry\;weight}{Saturated\;weight-Submerged\;weight}$$ 2

$$Total\;porosity\;\left(\%\right)=\left(1-\frac{\rho_{B}}{\rho_{TD}}\right)\times 100\%$$ 3

$$Open\;porosity\;(\%)=\frac{Saturated\;weight-Dry\;weight}{Saturated\;weight-Submerged\;weight}\times 100\%$$ 4

Closedporosity(%)=Totalporosity(%)−Openporosity(%) 5

The geometric bulk density (*ρ*_*GB*_) was determined from the dry weight and calculation of the volume from the mean of three measured values each of diameter (*d*) and height (*h*), as per Eq 6.


$$\rho_{GB}=\frac{4\times Dry\;weight}{\pi d^{2}}$$ 6

### 2.8 Morphology characterization

A Scanning Electron Microscope (SEM) (FlexSEM 1000, Hitachi, Japan) was used to image the fractured surfaces of sintered samples. Surface pore sizes were measured from the micrographs using image analysis software, Fiji [41], a distribution of ImageJ [42], as a measurement of Feret’s diameter of 100 locations per sample. The Feret’s diameter is the longest distance between any two points along the region-of-interest boundary [43].

### 2.9 Osteoblast attachment and proliferation

#### 2.9.1 Seeding and culturing of primary human osteoblasts

Normal primary human osteoblast cells (HOBs) were obtained from Lonza Biosciences (Walkersville, MD). The cells were cultured in Osteoblast Growth Medium Bulletkit (OGM, Lonza, USA), which contains basal medium, supplemented with 10 vol.% fetal bovine serum (FBS), 0.1 vol.% gentamicin/ amphotericin-B and 0.1 vol.% ascorbic acid. The HOBs were cultured at 37°C with 5% carbon dioxide and complete medium changes were performed every two to three days. All HOBs used in the experiments were at passage six. After the cells reached 80%-90% confluence, they were trypsinized with Trypsin/EDTA (Lonza, USA) and neutralized with Trypsin Neutralizing Solution (Lonza, USA), followed by centrifugation at 220g for 5 minutes to pellet the harvested cells. The cells were next diluted in OGM to a cell density of 5.6 × 10^5^ cells/ml. The specimens were placed in 24-well plates (Corning, Sigma Aldrich), which were pre-coated with Pluronic F-127 (Sigma Aldrich, USA) to reduce cell attachment to the wells. The HOBs were seeded on the disc specimens with an initial cell density of 6.4 × 10^4^ cells/cm^2^ per top surface area. The cell suspension was gently dripped onto the specimens and incubated for 90 min to allow for cell adhesion. Next, OGM was added to the wells at 3.3 × 10^4^ cells/ml, based on the number of initially seeded cells per specimen.

#### 2.9.2. Quantitative cell viability

HOB viability was evaluated after 7 days of culture, using the alamarBlue assay, a resazurin-based assay, for quantitation of metabolic activity (*n* = 3). Before the assay, the specimens were rinsed with PBS and transferred to new well plates to avoid measuring the metabolic activity of any cells that may be adhered to the well plate. Resazurin sodium salt (Sigma Aldrich, USA) in PBS at a concentration of 0.15 mg/ml was mixed with OGM at a ratio of 20:100 Resazurin solution to OGM. 1 ml of final working solution was placed in each well (including a blank well), followed by incubation for 4h. 100 μl each was transferred to a black well plate, and the absorbance was measured using a microplate reader (GloMax Explorer, Promega, USA) at the 560 nm wavelength. The results were normalized to the geometric surface area of the respective sample, calculated from the mean diameter of five replicates, with each replicate measured thrice.

#### 2.9.3. Confocal microscopy

Laser scanning confocal microscopy (A1R+, Nikon) was used to visually assess cell morphology and corroborate proliferation results. The scaffolds were rinsed and transferred to a new well plate. Each rinse was conducted with three washing cycles using PBS. The cells were fixed in 4 w/v% Formalin for 10 min at room temperature, then rinsed thrice with PBS. Permeabilization was conducted using 0.2 v/v% Triton X-100 solution in PBS for 10 min at room temperature. To block non-specific binding, the scaffolds were immersed in 1w/v% bovine serum albumin (BSA) (Sigma Aldrich, USA) solution in PBS for 1h. ActinRed 555 ReadyProbes reagent (Invitrogen, USA) for 30 min at room temperature was used to stain the F-actin in the cytoskeleton, then rinsed. To stain collagen Type I (extracellular matrix laid by the HOBs), cells were labelled with a rabbit anti-collagen I antibody (abcam, UK) at 1/250 dilution at 4°C overnight, followed by a rinse and a goat anti-rabbit secondary antibody with Alexa Fluor Plus 488 (abcam, UK) at 1/500 dilution for 1h at room temperature. The cell nucleus was counterstained with 4′,6-Diamidino-2-phenylindole dihydrochloride (DAPI) (Sigma Aldrich, USA) for 5 min at room temperature. After the final rinse, scaffolds were gently patted dry, then mounted to 8-well chambered coverglass (Nunc Lab-Tek II, ThermoFisher, USA) with SlowFade Antifade mountant (Invitrogen, USA). The scaffolds were imaged at 20× magnification in air.

### 2.10 Statistical analysis

All data are represented as the mean value ± standard error of the mean. A one-way analysis of variance (ANOVA), Fisher’s method for multiple comparisons, was used to evaluate differences among the groups. The Games-Howell method was used for the analysis of pore diameters where variances were not assumed equal amongst the groups. A p-value of < 0.05 is considered statistically significant. On the plots, a single asterisk (*) denotes p < 0.05, a double asterisk (**) denotes p ≤ 0.01, and a triple asterisk (***) denotes p ≤ 0.001. Statistical calculations were processed using the Minitab® Statistical Software [44]. All plots and charts were made with the Origin Pro software [45].

## 3. Results

### 3.1 Physical and chemical characterization

The median diameter, d_50_, of the β-TCP is 1.5 μm and 3.0 μm for the HA. The particle size distribution plots as well as other details can be referred to in the S1 Fig and S1 Table. The X-ray diffraction graph as presented in S2 Fig shows clearly that the remaining material after pyrolysis is β-tricalcium phosphate. The oil and CTAB surfactant were pyrolyzed without affecting the chemical composition of the β-tricalcium phosphate.

### 3.2. Density, porosity and mechanical behaviour

**Fig 3:**
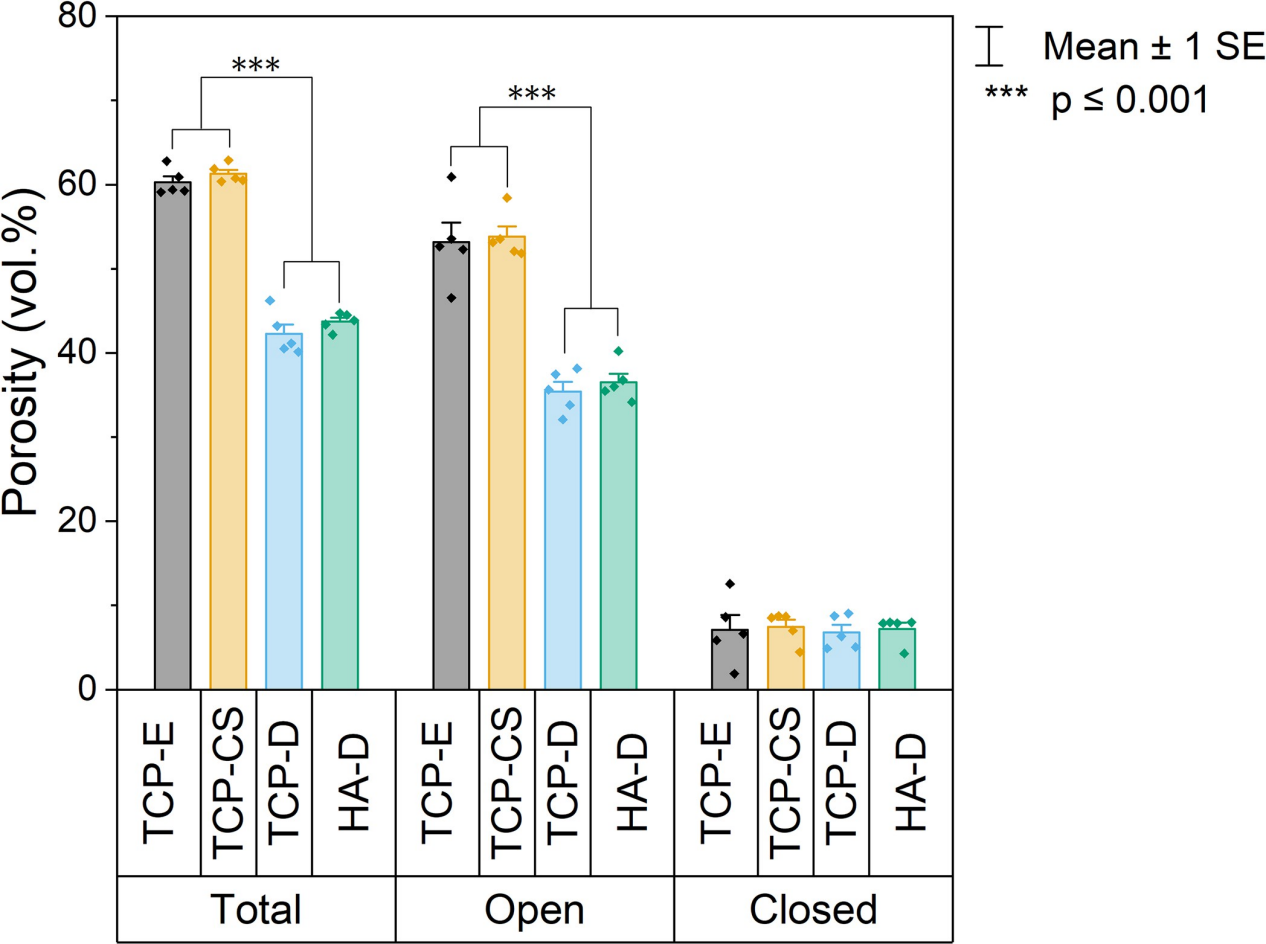
Means of percent of sample volume occupied by porosity in each formulation based on the Archimedes method, with error bars showing 95% confidence interval. Open porosity refers to pores which are accessible externally, whereas closed porosity refers to pores which are fully within the material without pore necks to the exterior. For total and open porosities, TCP-E and TCP-CS are statistically significantly higher than TCP-D and HA-D as indicated by the asterisks (p < 0.001). p > 0.05 for all closed porosities. (Total porosity in this figure = 100%—Archimedes bulk density %TD in Table *1*).

The sintered objects were cylinders of about 4 mm in height and 10 mm in diameter as shown in S3 Fig in the supplementary materials section. The sintered ceramic materials’ density and porosity was determined by geometric and Archimedes’ measurements as described above in Section 2.7. The densities of the materials are shown in Table 1. The materials were generally in the range of 40 to 60% of full density. The percentage of the volume which is porosity and the distribution of that porosity as either open or closed is given in Fig 3.

**Table 1:** Density data measured by the Archimedes and geometric methods. TD: theoretical full density.

Sample	Archimedes Apparent Density		Archimedes Bulk Density		Geometric Bulk Density	
	(g/cm^3^)	(% TD)	(g/cm^3^)	(% TD)	(g/cm^3^)	(% TD)
TCP-E (emulsion)	2.62	85.4	1.22	39.7	1.11	36.1
TCP-CS (capillary suspension)	2.58	84.0	1.19	38.7	1.05	34.2
TCP-D (dense)	2.75	89.5	1.77	57.8	1.64	53.6
HA-D (dense)	2.72	86.2	1.73	54.7	1.67	52.8

The porous TCP-E and TCP-CS have similar total porosities of (60.3 ± 0.7)% and (61.3 ± 0.5)% respectively. The dense TCP-D and HA-D have similar total porosities of (42.2± 1.1)% and (43.7 ± 0.5)% respectively. The open porosity for TCP-E is (53.2 ± 2.3)%, which is again close to that of TCP-CS at (53.8 ± 1.2)%. The open porosity for TCP-D and HA-D are lower at (34.4 ± 1.1)% and (36.5 ± 1.0)% respectively. The closed porosities for all four samples are not statistically different, with means ranging from 6.82% to 7.48%.

Not surprisingly, the formulations of β-TCP and HA without oil (TCP-D and HA-D in Fig 2) have a lower amount of overall porosity. The formulations with introduced porosity (TCP-E and TCP-CS) have means of total and open porosity which are statistically similar, and the dense formulations (TCP-D and HA-D) are similar as well, whereas the pairwise comparisons between samples in these two sub-groups (porous versus dense) are significantly different with p < 0.001. The means of closed porosity for all four samples are statistically similar. In other words, the materials processed with oil templating are lower in density and have more open pores accessible from the exterior surfaces than the dense structures.

The mechanical properties of the TCP-CS material are reported in ref 73 as compressive strength 1.39 MPa, flexural strength 1.09 MPa, compressive modulus, 26 MPa and flexural modulus 440 MPa. The mechanical properties of the TCP-E and TCP-D materials are estimated as described in the supplementary materials section and reported in S2 Table. The mechanical properties of TCP-E are about twice those of TCP-CS while the mechanical properties of TCP-D are significantly higher at about 10 to 20 times those of TCP-CS. These strength and modulus values are within the reported range of trabecular bone (strength ranging around 0.5–30 MPa [46, 47] and an elastic modulus of about 20–5000 MPa [16]).

### 3.3 Pore morphology and size

**Fig 4:**
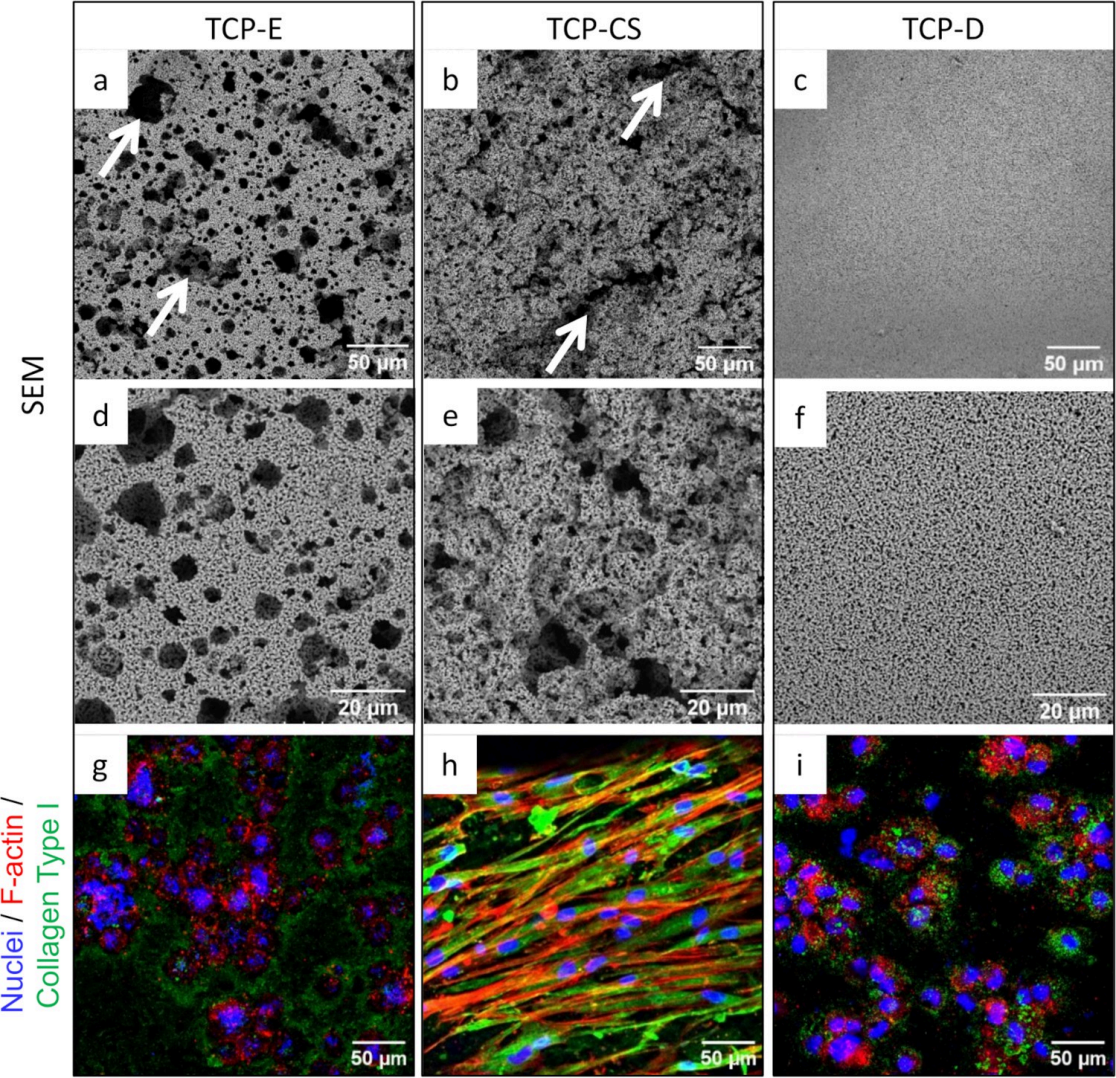
(a-f) Scanning electron micrographs of the as-fired surfaces of the β-TCP formulations at two levels of magnification. (a and d) TCP-E (emulsion) displays mainly spherical pores (highlighted by white arrows), whereas (b and e) TCP-CS (capillary suspension) has an uneven surface comprising elongated channels (highlighted by white arrows) and smaller pores. (c and f) The surface of TCP-D (dense) is generally flat with largely submicron-sized pores. (g, h, i) Confocal microscopic images with osteoblasts identified by the nuclei (blue), F-actin cytoskeleton (red) and the collagen type I (green) extracellular matrix.

The differences in pore morphology across the formulations are apparent in the electron microscopy images presented in Fig 4. The emulsion formulation (TCP-E, Fig 4A and 4D) produces pores that are formed by individual oil droplets so that the pore shapes are somewhat like spheres [34, 39, 48] on order of 5 to 20 microns. Whereas the capillary suspension formulation (TCP-CS, Fig 4B and 4E) produces pores of unequal aspect ratios because the oil exists in the suspension as capillary bridges between particles. Although it is difficult to estimate the length of the channels, they appear to be on order of 20 to 100 microns. In both samples created from formulations with introduced porosity (see Fig 4D for TCP-E, and Fig 4E TCP-CS), there appears to be ‘windows’ on the walls of the pores. This suggests that there is an interconnected pore network where pores are connected by these ‘windows’, and internal pores possibly accessible from the exterior *via* these networks. An image of a dense sample produced without added oil (TCP-D) is also included in Fig 4C and 4F for comparison, which shows only submicron-scale porosity from necking between particles during sintering. The general surface topography for TCP-CS is much more varied than that for TCP-E and TCP-D.

**Fig 5:**
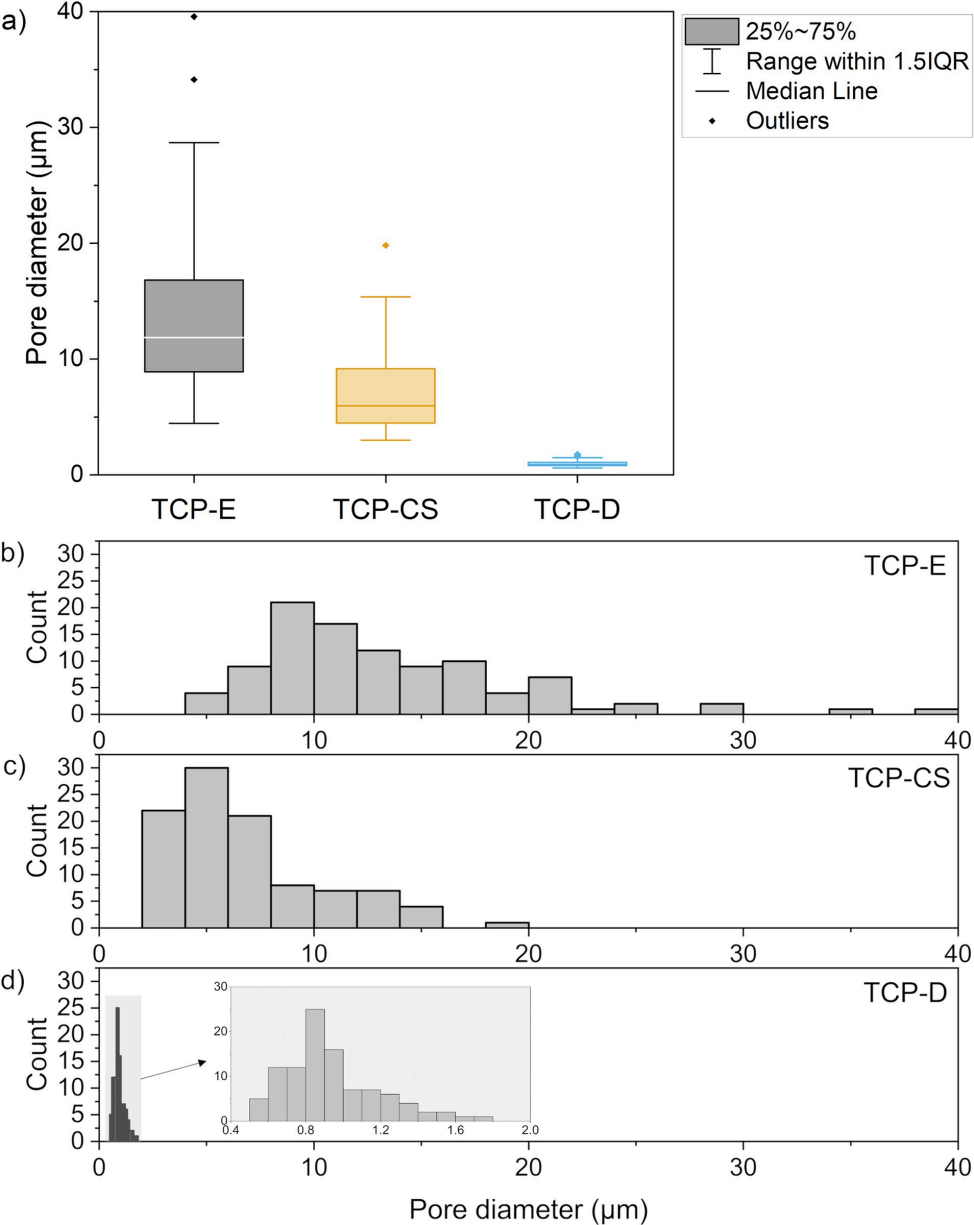
(a) Box plots of pore diameters–box indicates the 25% to 75% confidence interval, with the median line in the middle; whiskers show the 1.5 interquartile range; and dots beyond the whiskers denote outliers. (b-d) Histograms showing distribution of pore diameters of (b) TCP-E, (c) TCP-CS, and (d) TCP-D (with inset at a different scale). Games-Howell post-hoc test revealed that all three samples are statistically significantly different from each other with p < 0.001 for all pairwise comparisons.

Fig 5 show the median and distribution of pore size in each of the TCP samples (TCP-E, TCP-CS and TCP-D). The pore sizes amongst the TCP samples with varying microstructures show a statistically significant difference with each other, all pairwise comparisons revealing a p-value of less than 0.001. The pores in the emulsion (TCP-E) are the largest, averaging (13.50 ± 0.61) μm. The dense suspension (TCP-D) has the smallest pores of (0.98 ± 0.03) μm diameter, within expectations as it does not have oil porogens to act as soft templates for pores. Between these two extremes, lie the capillary suspension (TCP-CS) which has pore diameters of (6.84 ± 0.23) μm. Do note that the elongated channels in TCP-CS are not included in these measurements, as they meander, and it is difficult to ascertain their starting and ending points. We believe that the larger oil droplets (seen in the emulsion) are not stable with increased surfactant and oil. Instead, the oil forms capillary, pendular and perhaps funicular bridges between the particles which could produce capillary suspensions [49–52], perhaps associated with primary phase inversion [40, 53]. Another possibility is the formation of a bijel structure, but under these circumstances, the particles would remain primarily in the aqueous phase [36, 37]. These bridged structures form the channels visible in Fig 4B.

### 3.4 *In vitro* performance of osteoblasts on scaffolds

**Fig 6:**
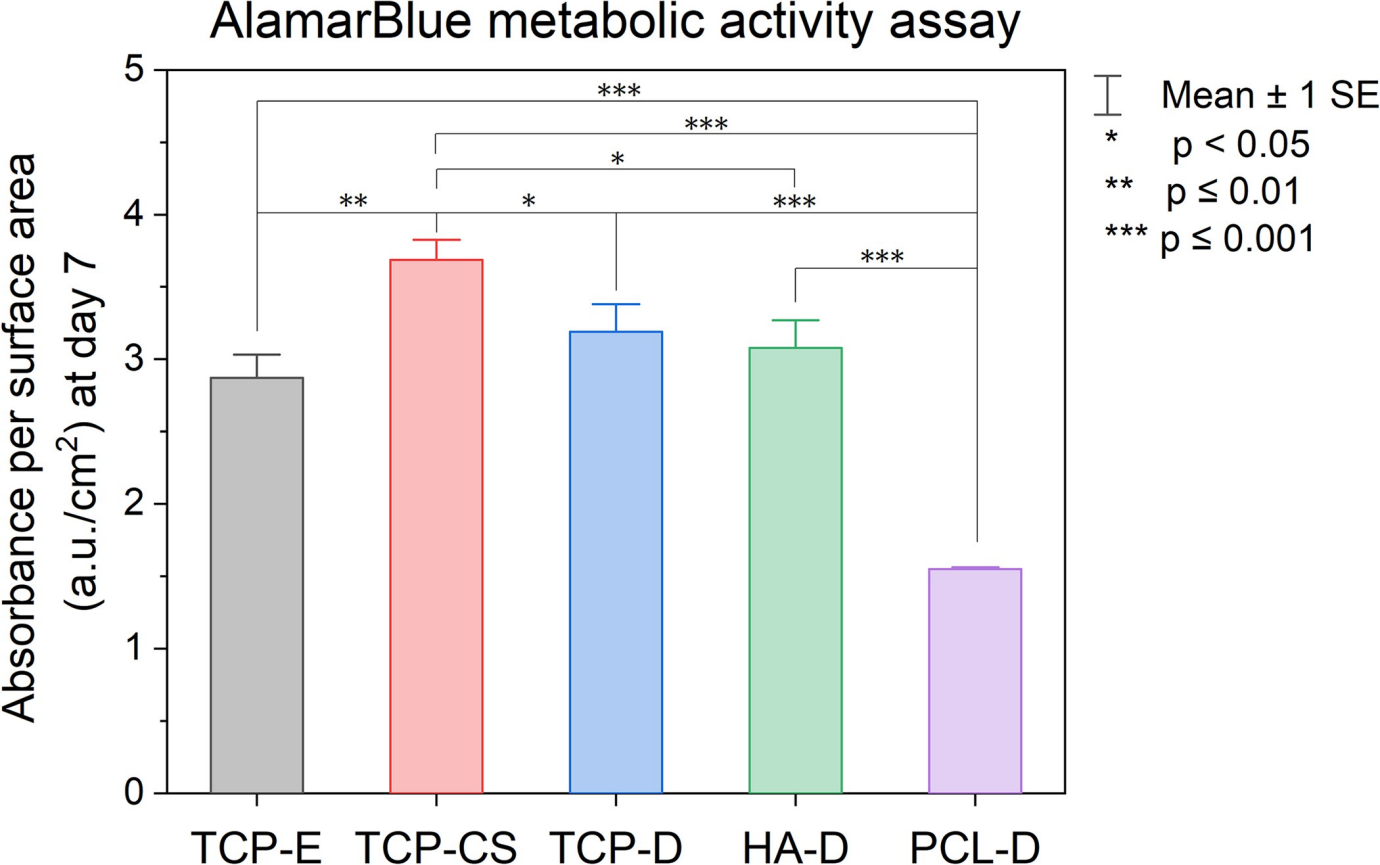
Graph of metabolic activity of human osteoblasts after 7 days of incubation on scaffolds of varying microstructure (TCP-E, TCP-CS and TCP-D) as well as different materials without templated porosity (TCP-D, HA-D and PCL-D). Solid bars illustrate the means and error bars represent the standard error. All pairs show a statistically significant difference, except for TCP-E and TCP-D, TCP-E and HA-D, as well as TCP-D and HA-D.

Fig 6 shows the metabolic activity of human osteoblasts on the scaffolds after 7 days of *in vitro* culture, which is used as a proxy for cell number. All pairwise comparisons reveal a statistically significant difference except for TCP-E and TCP-D, TCP-E and HA-D, as well as TCP-D and HA-D. When making a comparison of the three materials– β-TCP, HA and PCL–without the larger templated pores, TCP-D and HA-D show similarly high metabolic activity, both with a statistically significant increase from PCL-D. Therefore, β-TCP and HA appear to provide a more conducive environment for osteoblasts than PCL. When compared to PCL-D as a control, all of the bio-ceramic samples, regardless of TCP or HA, with or without porous microstructure, facilitate significantly higher cell viability than PCL. Next, we compare the cell viability on the three TCP microstructures (TCP-E, TCP-CS and TCP-D). We observed that the type of porosity significantly impacted the osteoblast response. The highest metabolic activity was recorded for TCP-CS, whereas TCP-E appeared to have the lowest level of metabolic activity. TCP-D was found to perform worse than TCP-CS but similar to TCP-E.

**Fig 7:**
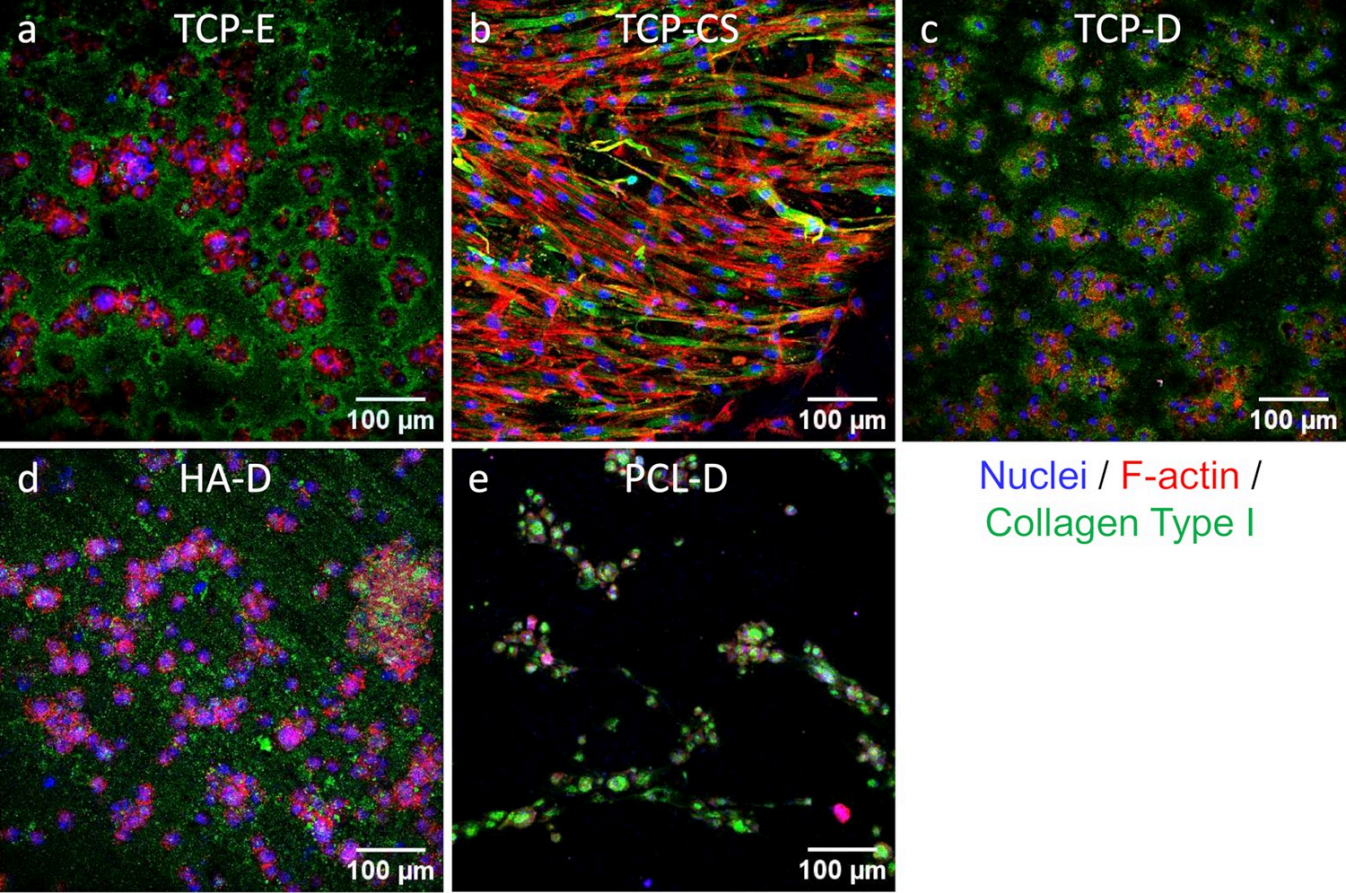
Confocal microscopy of osteoblasts on the surfaces after 7 days of incubation. (a) TCP-E, (b) TCP-CS, (c) TCP-D, (d) HA-D and (e) PCL-D. Blue represents the cell nucleus; red dyes the F-actin cytoskeleton of the cell; green indicates the presence of Collagen Type I which is extracellular matrix laid down by viable cells.

The confocal images in Fig 7 are a visual indication of the anchorage and spread of osteoblasts on the sample surfaces. The osteoblasts are found mainly within the pores of TCP-E. The cells are observed to be significantly elongated in TCP-CS, a marked difference from the rounded cells found on the dense TCP-D, HA-D and PCL-D. TCP-D and HA-D show a more homogeneous distribution of osteoblasts across the surface than the aggregated cells seen on PCL-D. In terms of quantity, it is a visual confirmation of the assay results in Fig 6 that there are more cells on the TCP and HA scaffolds than on the PCL. Collagen Type I was also visualised in the confocal micrographs, as it is a main component of bone and important in the bone remodelling process. All surfaces exhibited some degree of collagen staining, though notably, the TCP surfaces showed the most robust collagen staining. Interestingly, the staining was centralised around the clusters of cells on the TCP-D and TCP-E surfaces, while it was more evenly present on the TCP-CS surfaces.

## 4. Discussion

### 4.1 Influence of formulation on surface pore morphology

The 10 micrometre-scale, nearly spherical surface pores seen in Fig 4A and 4D are typical of particle-stabilized emulsions (also known as Pickering emulsions), where the hydrophobized solid particles decorate around the oil droplets at the oil-water interface and stabilize the droplets as spheres. With an increase in surfactant adsorbed to the β-TCP particles, the hydrophobicity of the particles is increased, consistent with previous observations [40, 53]. When the contact angle at the oil-water interface increases beyond the optimal angle to form particle-stabilised emulsion droplets, it is possible that phase inversion occurs and the particles move into the oil phase. Especially at a higher oil fraction, the oil forms bridges between particles to create a particle network with bicontinuous interpenetrating oil and aqueous phases which produce channels between the particles [39, 50, 51]. The channels formed by the individual phases act as soft templates leaving behind elongated pores after pyrolysis, as described in Fig 1 and observed in Fig 4B and 4E. Both the nearly spherical pores and elongated channels appear to account for the additional amount of open porosity in Fig 3, which is beneficial for cell penetration and migration.

### 4.2 Osteoblast viability on different surface pore morphology

In the emulsion (TCP-E, Fig 4G), the cells appear to the authors to be associated with the surface pores because of the similar size and shape. The surface pores with cells in Fig 4G appear slightly larger than the pores before cell culture (Fig 4A), suggesting that the pores may have been enlarged by resorption [54] or perhaps the cells were involved in the ceramic resorption [55–57]. These cells appear to be primarily equiaxed in shape. That is a promising sign of cell migration into the scaffold, which is important for the formation of bone tissue throughout the scaffold and eventually replacing the scaffold. It has been evidenced by others that greater porosity increases the osteoinductivity of the scaffold as well as bone grows within pores <50 μm and even <10μm in diameter [58]. So it is surprising that despite TCP-E having significantly more porosity than TCP-D (Fig 3) as well as having suitable surface pore sizes (Fig 5A and 5B), it did not demonstrate a higher cell viability than the dense scaffold.

The mean cell viability of TCP-E appears to be slightly lower than TCP-D, however they are not statistically different (Fig 3). Increased microporosity has been shown elsewhere to improve new bone formation [58, 59], possibly due to increased surface area for cell adhesion [59] and increased protein adsorption [60, 61]. However, these studies compare different sizes or volume fractions of microporosity instead of against a sample void of microporosity as in this study. It is possible that the cells settled into the surface pores of TCP-E, which act like isolated pockets with reduced fluid flow. On the other hand, the cells adhered to the flat surfaces of TCP-D have increased access to nutrients, oxygen and waste diffusion. Despite the cells in TCP-CS also settling into the interior of its pores, the authors speculate that its longer channels may offer overall more nutrient flow than the sphere-like pores of TCP-E. As mentioned in section 3.3, the lengths of the elongated channels of TCP-CS are difficult to determine exactly but are typically much longer than the diameters of the sphere-like pores of TCP-E, thus resulting in increased connectivity to the exterior. Additionally, the morphological improvements of TCP-CS to cell growth as discussed below may have overruled any reductions from this and resulted in an overall increase over that of TCP-E.

Constrictions from the interconnection windows between pores or pores to the exterior can lead to localized accumulation of ions in the micropores and an ion gradient, which promotes mineralization and osteoblast migration [62, 63]. Possibly there is a minimum requirement of micropore interconnection size for bone formation [62], which TCP-E did not meet whereas channels of TCP-CS can be thought of as a series of micropores with connection widths approximately the diameter of the micropores.

The osteoblasts cultured on the capillary suspension (TCP-CS, Fig 4H) have a highly elongated morphology. Their growth is likely to be following the surface channels produced by the oil-water phase inversion and separation as indicated by the arrows in Fig 4B. Such a cellular spreading or extension of cytoplasmic processes demonstrates good adhesion to the substrate [64, 65]. Our results support previous studies that show anisotropy of the surface topography (or grooved surfaces) enhances osteoblastic proliferation [21, 22], as well as the synergistic effect of microscale and sub-micron roughness [24]. The ability to control cell shape and morphology by varying the surface pore morphology is an important outcome of this research. Furthermore, this relatively cost-effective fabrication method of an interconnected pore network (of about 53% of sample volume as presented in Fig 3) is an additional advantage for enhanced bone ingrowth [66] over widely studied methods of surface modification, such as grinding [21, 22], electron beam irradiation [23], anodization and chemical etching [24].

Other fabrication methods of porous scaffolds have been proposed, such as (i) by the introduction other porogens such as air (foaming); or (ii) from the architectures created by additive manufacturing or 3D printing processes. 3D-printing by direct ink writing typically forms cylindrical filaments, which have convex surfaces. In comparison, air bubbles in foams or oil droplets in emulsions form pores with concave surfaces. Only the concave surfaces of calcium-deficient hydroxyapatite foams were reported by Barba et al. to induce significant ectopic bone formation, and not the 3D-printed scaffolds with convex pores [67]. Similarly, 3D-printed hydroxyapatite scaffolds were found by Liu et al. [68] to promote bone marrow stem cell spreading and a higher cell density when 3D-printed with emulsions as compared to a dense suspension. This enhancement was attributed to the additional adhesion sites for cells provided by the microporous filament surfaces. Despite the merits of pores formed by foams or emulsions, they are commonly limited to relatively simple overall shapes and homogeneous internal structures [69]. On the other hand, 3D-printing is advantageous in its ability to create near-net shapes, which can be easily customised to the implant site. Also, the precise control of 3D printing holds potential in creating functionally graded scaffolds to further tailor the mechanical properties or degradation behaviour of BTE scaffolds [70–72].

The volume percent of total and open porosities in TCP-E and TCP-CS were very similar (Fig 3), so the difference in their cell viability numbers could possibly be attributed to the different surface pore morphologies. The capillary suspension (TCP-CS) showed the highest level of metabolic activity of all the samples (Fig 6), which could be due to cellular spreading on the surface promoting cell growth [21, 22]. As compared to its dense counterpart of the same material (TCP-D), the mean metabolic activity of TCP-CS was 16% higher. When compared to the dense substrates of other materials, HA-D and PCL-D, TCP-CS was 20% and 138% higher respectively. In contrast, for the dense substrates (Figs 4I and 7C–7E), regardless of material (TCP-D, HA-D and PCL-D), the cells maintain their round shapes with negligible spreading. Osteoblasts *in vivo* display a characteristic cuboidal or polygonal structure, and can differentiate into osteocytes [73, 74]. Osteocytes are interconnected stellate cells with dendritic or cytoplasmic processes [75, 76]. Surface topography or roughness (within a scale perceivable by the cell) has been shown to have a positive effect on cell differentiation, as evidenced by others [77–79].

The *in vitro* cellular responses in this study demonstrate that the surface pore morphology of the emulsion and capillary suspension provide suitable 3D topographical conditions to the cells, promoting cell migration on the surface and adhesion to the surface respectively. Furthermore, the fabrication method proposed of using oil as a porogen is economical and its removal during sintering is straightforward. The particle-stabilised emulsions created in this study were stable for long periods of time–phase separation was not observed even after 2 to 3 months, simply stored in containers at ambient conditions. This long-term stability has also been reported in other work, attributed to the adsorption of colloidal particles at the gas-liquid (foams) or liquid-liquid (emulsion) interfaces that promote stabilization and inhibit coalescence and Ostwald ripening [48]. The pore sizes from air bubbles in foams tend to be larger than that from oil droplets in emulsions. An advantage of using emulsions is that the oil droplets are less compressible or deformed under pressure than air bubbles. This can be beneficial for subsequent processing, such as to reduce die swell and prevent collapse when shear stresses are applied during direct ink writing, a material extrusion 3D printing technique.

### 4.3 Osteoblast response on different materials

PCL is a popular synthetic polymer used as a base material for 3D-printed bone tissue scaffolds [80–83], and therefore was chosen as a control for this work. Our hypothesis that β-TCP and HA will perform better is supported by the results in Fig 6 that there is significantly more metabolic activity in both bio-ceramics than in PCL. Furthermore, it is corroborated visually in the confocal images in Fig 7. Due to PCL’s hydrophobic nature, poor cell adhesion to the scaffold results; PCL would require further treatment to improve its osteoconductivity [83–85].

β-TCP performs slightly better than HA in terms of cell metabolic activity, thus it is selected as the main material for further investigation. Our findings corroborate current knowledge using other models: greater *in vivo* proliferation was induced with β-TCP than HA [86] or biphasic calcium phosphate (BCP) than HA [87], as well as increased *in vitro* mesenchymal stem cell proliferation in TCP compared to a composite of HA/TCP [88]. This work provides a foundation to build upon for producing 3D tissue scaffolds with an additional level of macroporosity *via* the filament spacing by Direct Ink Writing (DIW, an extrusion-based Additive Manufacturing technique). Further investigation of mechanical performance, influence of degradation, and extended *in vitro* performance of osteoblasts was in progress at the time of writing. The current work suggests that capillary suspensions should be the preferred paste formulation to produce surface pore morphology to maximize osteoblast growth.

## 5. Conclusions

Utilizing oil for soft templating, different porous surface microstructures (emulsion and capillary suspension) were created in bio-ceramics by varying the oil and surfactant concentrations. The nearly spherical surface pores of the emulsion and the elongated surface channels of the capillary suspension form differing physical interfacial environments, which greatly influence the behavior of osteoblastic growth. In terms of material, metabolic activity was found to be highest in beta-tricalcium phosphate (β-TCP) and hydroxyapatite (HA), compared to polycaprolactone (PCL). Amongst the surface pore morphologies, the capillary suspension demonstrated enhanced cell proliferation, followed by the non-porous structure. The β-TCP capillary suspension scaffold (TCP-CS) provided the most favorable conditions in response to osteoblasts, including cell spreading. After 7 days of culture, TCP-CS demonstrated a 16% increase in metabolic activity over a dense scaffold of the same material (TCP-D), a 20% increase over hydroxyapatite (HA-D) and an exceptional 138% increase over PCL. Therefore, the β-TCP capillary suspension scaffold (TCP-CS) shows promise as a potential candidate in bone regeneration applications. Furthermore, the fabrication strategy proposed creates economical and stable suspensions, which produce controllable, micrometer-scale pores with interconnected porosity.

## Supporting information

S1 Fig Particle size distribution of a) β-tricalcium phosphate (β-TCP) powders, and b) hydroxyapatite (HA) powders.
(PDF)

S2 Fig X-ray diffraction results of β-TCP formulations after sintering, showing no change to the composition as additives are burnt out during calcination. (PDF)

S3 Fig Photographs of a) TCP-E sintered cylinders; b) TCP-CS sintered cylinders; c) TCP-D sintered cylinders and d) HA-D sintered cylinders.
(PDF)

S1 Table Particle size statistics of β-TCP and HA powders used for this study. (PDF)

S2 Table Values of strengths and elastic moduli for materials tested under both uniaxial compression as well as 3-point bending (mean ± standard error). * measured values as reported in reference 73. # estimated by ratio between 3D printed scaffolds of same material reported in reference 73.
(PDF)
