# Supplementary material for: Tailoring the surface pore morphology of bioceramic scaffolds through colloidal processing for bone tissue engineering
Source: PLoS One. 2025 Feb 27;20(2):e0318100. doi: 10.1371/journal.pone.0318100 (PMC11867385; doi:10.1371/journal.pone.0318100)
Supplement: S1 Fig — Particle size distribution of a) β-tricalcium phosphate (β-TCP) powders, and b) hydroxyapatite (HA) powders. (PDF) [file pone.0318100.s001.pdf]

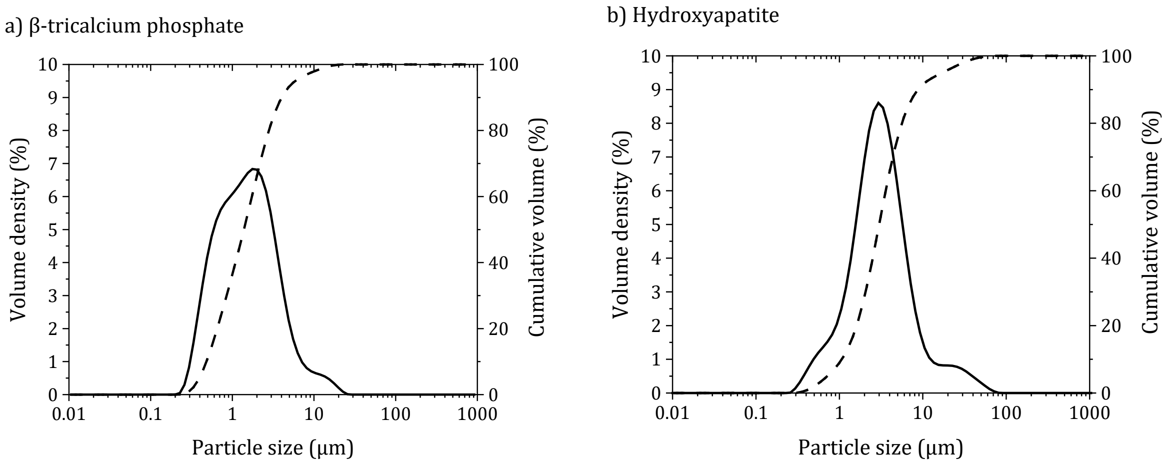

Figure S1. *Particle size distribution of a) β-tricalcium phosphate (β-TCP) powders, and b) hydroxyapatite (HA) powders.*
