# Supplementary material for: Tailoring the surface pore morphology of bioceramic scaffolds through colloidal processing for bone tissue engineering
Source: PLoS One. 2025 Feb 27;20(2):e0318100. doi: 10.1371/journal.pone.0318100 (PMC11867385; doi:10.1371/journal.pone.0318100)
Supplement: S2 Fig — (PDF) [file pone.0318100.s002.pdf]

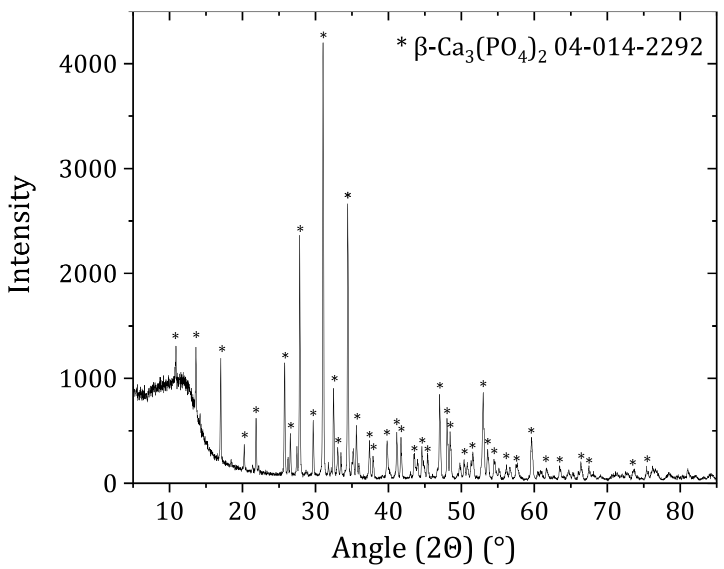

Figure S2. *X-ray diffraction results of β-TCP formulations after sintering, showing no change to the composition as additives are burnt out during calcination.*
