# Supplementary material for: Tailoring the surface pore morphology of bioceramic scaffolds through colloidal processing for bone tissue engineering
Source: PLoS One. 2025 Feb 27;20(2):e0318100. doi: 10.1371/journal.pone.0318100 (PMC11867385; doi:10.1371/journal.pone.0318100)
Supplement: S3 Fig — Photographs of a) TCP-E sintered cylinders; b) TCP-CS sintered cylinders; c) TCP-D sintered cylinders and d) HA-D sintered cylinders. (PDF) [file pone.0318100.s003.pdf]

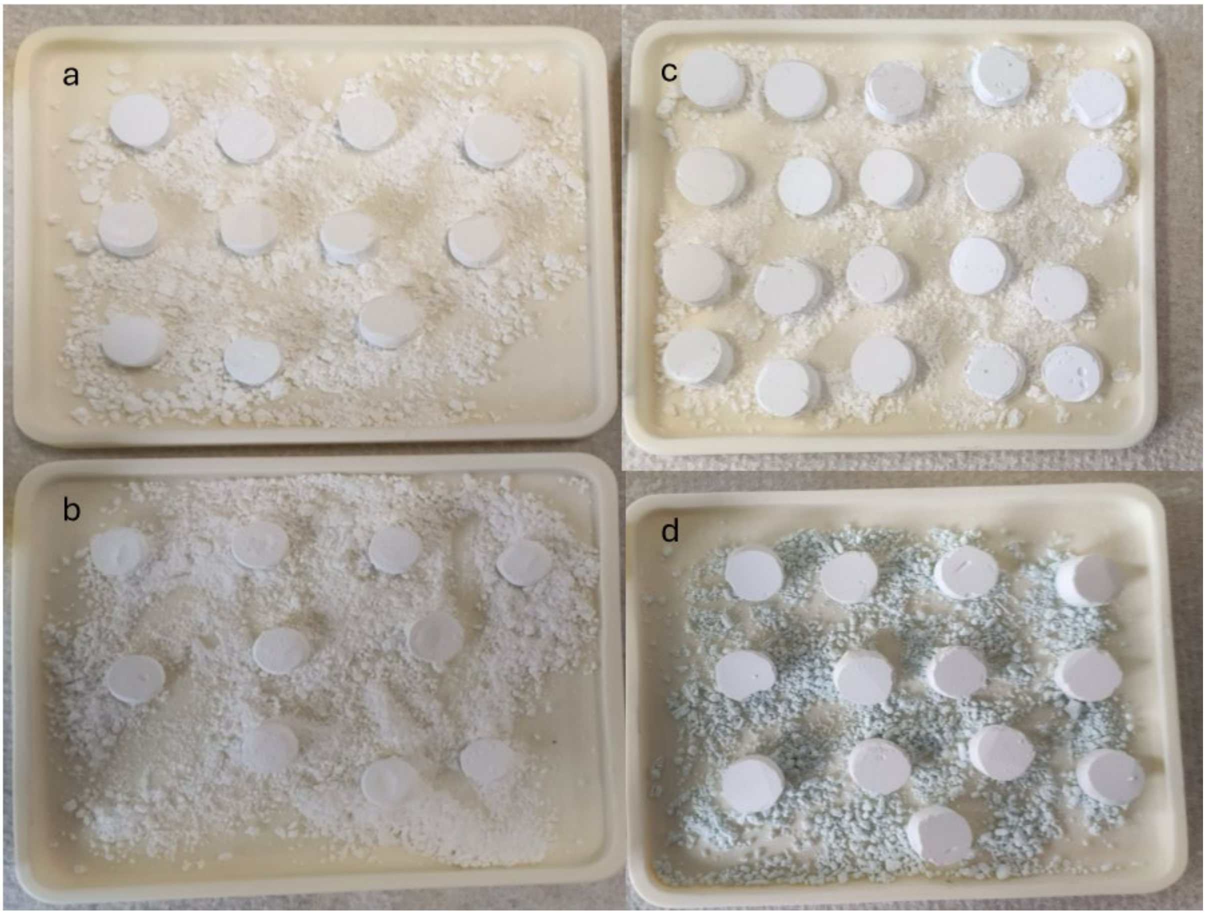

Figure S3. *Photographs of a) TCP-E sintered cylinders; b) TCP-CS sintered cylinders; c) TCP-D sintered cylinders and d) HA-D sintered cylinders.*
