# Supplementary material for: Tailoring the surface pore morphology of bioceramic scaffolds through colloidal processing for bone tissue engineering
Source: PLoS One. 2025 Feb 27;20(2):e0318100. doi: 10.1371/journal.pone.0318100 (PMC11867385; doi:10.1371/journal.pone.0318100)
Supplement: S1 Table — (PDF) [file pone.0318100.s004.pdf]

	d_{10} (µm)	d_{50} (µm)	d_{90} (µm)
**β-TCP**	0.51	1.48	4.31
**HA**	1.06	3.00	8.76

Table S1. *Particle size statistics of β-TCP and HA powders used for this study.*
