# Supplementary material for: Tailoring the surface pore morphology of bioceramic scaffolds through colloidal processing for bone tissue engineering
Source: PLoS One. 2025 Feb 27;20(2):e0318100. doi: 10.1371/journal.pone.0318100 (PMC11867385; doi:10.1371/journal.pone.0318100)
Supplement: S2 Table — * measured values as reported in reference 73. # estimated by ratio between 3D printed scaffolds of same material reported in reference 73. (PDF) [file pone.0318100.s005.pdf]

We have estimated the mechanical behaviour of the TCP-E and TCP-D using the ratio of the slip cast property to the 3D printed scaffold property as published in ref 73. For example, the compressive strength, σ, of the slip cast TCP-E is estimated as follows:

$$\frac{\sigma_{capillary\ suspension-slipcast}}{\sigma_{capillary\ suspension-DIW}} = \frac{\sigma_{emulsion-slipcast}}{\sigma_{emulsion-DIW}}$$

$$\frac{4.32\ MPa}{2.37\ MPa} = \frac{\sigma_{emulsion-slipcast}}{1.39\ MPa}$$

$$\sigma_{emulsion-slipcast} = 2.5\ MPa$$

**Sample**	**Uniaxial Compression**		**Flexural 3-point Bending**	
	**Strength (MPa)**	**Young's Modulus (MPa)**	**Strength (MPa)**	**Elastic Modulus (MPa)**
Slip cast TCP Capillary Suspension*	1.39 ± 0.31	26 ± 4	1.09 ± 0.10	440 ± 55
Slip cast TCP Emulsion#	2.5	67	2.2	719
Slip cast TCP Dense#	23.5	1040	12.3	4086

Table S2. *Values of strengths and elastic moduli for materials tested under both uniaxial compression as well as 3-point bending (mean ± standard error).*

** measured values as reported in reference 73.*

# estimated by ratio between 3D printed scaffolds of same material reported in reference 73.
